# Propelling Nurse-Led Structured Intervention to Enhance Self-Care among Patients with Chronic Heart Failure (PROACT-HF): A Cluster Randomized Controlled Trial Study Protocol

**DOI:** 10.3390/jpm14080832

**Published:** 2024-08-06

**Authors:** Momoko Okazaki, Takahiro Suzuki, Atsushi Mizuno, Toshimi Ikegame, Noriki Ito, Mai Onoda, Ikuko Miyawaki, Yuka Moriyama, Taku Yabuki, Satomi Yamada, Daisuke Yoneoka, Yuko Iwasawa, Kyoko Tagami, Kumiko Yoshikawa

**Affiliations:** 1 Department of Cardiovascular Medicine, St. Luke’s International Hospital, Tokyo 104-8560, Japantasuzuki@luke.ac.jp (T.S.); 2 Leonard Davis Institute for Health Economics, University of Pennsylvania, Philadelphia, PA 19104, USA; 3 Department of Nursing, Sakakibara Heart Institute, Tokyo 183-0003, Japan; 4 Department of Nursing, Yumino Medical Corporation, Tokyo 171-0033, Japan; ito@yumino-clinic.com; 5 Department of Nursing, Social Insurance Union of Societies Related to Nursing, Tokyo 150-0001, Japan; 6 Department of Nursing, Graduate School of Health Sciences, Kobe University, Kobe 654-0142, Japan; nsikuko@kobe-u.ac.jp; 7 Department of Nursing, Aso Iizuka Hospital, Fukuoka 820-8505, Japan; ymoriyamah1@aih-net.com; 8 Department of Internal Medicine, Tochigi Medical Center, Tochigi 320-8580, Japan; tyabu7973@hotmail.com; 9 Department of Medicine, Kawasaki University of Health and Welfare, Okayama 701-0193, Japan; satomiy@hp.kawasaki-m.ac.jp; 10 Center for Surveillance, Immunization, and Epidemiologic Research, National Institute of Infectious Diseases, Tokyo 162-8640, Japan; yoneoka@niid.go.jp; 11 Department of Nursing, Japanese Nursing Association, Tokyo 150-0001, Japan

**Keywords:** heart failure, self-care, nurse-led structured intervention, European Heart Failure Self-Care Behavior Scale

## Abstract

Background: Heart Failure (HF) is a common chronic disease that has a high readmission rate and is associated with worsening symptoms and major financial impacts. Disease management implemented during or after an HF hospitalization has been shown to reduce hospitalization and mortality rates. Particularly for outpatients, it is necessary to provide self-care interventions. Structured nurse-led support such as timely follow-ups, including phone calls, is beneficial for improving self-care assessments. Evidence for nurse-led support has been investigated but is less than conclusive. The aim of this study is to compare the effectiveness of a nurse-led structured intervention for outpatients with chronic HF against the usual medical care in terms of self-care behaviors and occurrence of symptom exacerbation or rehospitalization. Methods and analysis: This is a cluster-randomized controlled trial. A total of 40 facilities with certified HF nurses will be allocated to two-arm clusters at a 1:1 ratio, randomly to the intervention or usual care arms. A total of 210 participants will be assigned from the hospital. Participants will be adults aged 18 years or older diagnosed with chronic HF who are classified as Stage C according to the ACCF/AHA Heart Failure staging system. In the intervention group, patients will receive structured nursing support. This begins with weekly support, including phone calls, for the first month, then transitions to monthly support thereafter. The aim is to ensure the stability of their living conditions, promote medication adherence, and encourage self-management. In the control group, patients will receive the usual care. Primary outcomes will assess the improvement or continuation of self-care behavior as measured by changes in EHFScBS (European Heart Failure Self-Care Behavior Scale) scores. Secondary outcomes include occurrence of readmission within 30 days, 3 months, 6 months, and 1 year after discharge, duration of home care until readmission, and blood levels of BNP and NT-proBNP.

## 1. Introduction

### Rationale

Heart failure (HF) is a prevalent cause of mortality worldwide and presents a considerable financial burden through repeated hospitalization expenses. This issue is a global problem. As many guidelines recommend, despite the recent advancements in drug therapy for HF, it is crucial to acknowledge the complementary role of non-pharmacological interventions in order to optimize patient outcomes [1,2]. Among non-pharmacological interventions, self-care interventions is crucial for maintaining physical and emotional stability, promoting overall health, and reducing the burden on healthcare systems, as highlighted by the European Society of Cardiology guidelines [3]. Evidence has shown that self-care interventions are particularly beneficial, as they can reduce hospitalization and mortality rates, improve quality of life, and increase knowledge about the condition.

Despite the importance of self-care interventions, there is still no consensus on the optimal approach for promoting and enhancing self-care among individuals with HF. One promising strategy is a multidisciplinary HF disease-management program that includes structured support such as timely follow-ups, including phone calls, which have been shown to be effective in improving outcomes [4]. Previous systematic reviews have demonstrated that structured telephone support has reduced all-cause mortality and HF-related hospitalizations [5,6]. Based on these results, the 2013 ACCF/AHA HF clinical practice guideline and 2019 ACC Expert Consensus Decision Pathway have described follow-up phone calls have been recommended within 72 h after admission to improve outcomes [7,8]. The Heart Failure Association of the European Society of Cardiology and some Asian cardiac societies also suggest that phone calls can theoretically assess patient symptoms, improve adherence, and lead to timely treatment. However, structured self-care interventions, including early follow-up phone calls within 72 h, require significant additional human resources and costs, resulting in limited usage of these interventions [9,10]. Despite the theoretical potential for effectiveness, research is lacking, particularly regarding the additive effectiveness of early structured telephone support [11,12]. Therefore, the effectiveness of additional early structured telephone support interventions should be re-evaluated.

Among these multidisciplinary team members, nurses are integral members, because nurse-led self-care interventions have been considered effective and cost-effective to improve cardiovascular disease, and offer numerous benefits, including a favorable perception of nurses as trusted healthcare professionals, a high quality of care delivery provided by these experts, a more frequent and consistent follow-up provided to patients, and the potential for lower healthcare expenditures [13,14]. Implementation of structured self-care interventions including early telephone support, especially by nurses, could effectively improve self-care behavior and prevent symptom deterioration among HF patients. Additionally, the intervention promotes early medical attention and shows preventive effects against worsening symptoms during unscheduled visits.

The aim of the study described in this protocol is to conduct a cluster RCT to compare the clinical effectiveness of early nurse-led structured telephone support intervention with the usual care. In addition to assessing cardiovascular outcomes, such as all-cause death and HF rehospitalization, the study will also evaluate the effectiveness of the interventions through self-care assessments, considering the mechanism of the intervention. Overall, this study could provide valuable evidence on the effectiveness of nurse-led telephone interventions in improving self-care behavior and outcomes for HF patients.

## 2. Methods and Analysis

### 2.1. Trial Registration

The trial is registered with the Japan registry of clinical trials (ID:1030220447). Trial registration was completed before participant recruitment. Modifications to the protocol will be updated online.

### 2.2. Trial Design

We will conduct a cluster randomized controlled trial across different facilities to compare the efficacy of outpatient support by telephone and in person, as provided by a nurse.

### 2.3. Sample Size

A sample size of 210 (105 in each arm) will allow us to detect a difference of four units in the EHFScBS scores with 80% statistical power. Assumptions are based upon data from the CHART-2 study, which estimated a mean clinical estimated difference of 4 in the EHFScBS before and after intervention, with a standard deviation of 8.5 [15,16]. The ICC was 0.05, and a dropout rate of 30% due to mortality or other reasons over one year was assumed, based on previous research results, in order to determine the sample size [17,18].

### 2.4. Study Locations

Eligible facilities will be determined based on whether they provide training for cardiovascular specialists certified by the Japanese Circulation Society, and their regional characteristics. The centers for this study will be hospitals or clinics that provide both inpatient and outpatient care for patients with chronic heart failure (HF). They must meet the following criteria:

1. They must employ one or more nurses with expertise in chronic disease nursing, and who is certified as a specialist in chronic HF nursing or as an HF nurse.
2. They must be able to establish a system for providing both in-person and telephone-based support for chronic HF patients in the outpatient setting, led by certified HF nurses or nurses with more than 3 years of experience in HF nursing.

Potential intervention participants who meet the selection criteria for participation in the study will receive both written and oral explanations of the purpose and content of the study from the collaborating research institution. Patients who express interest in participating will be asked to sign a consent form. Once practitioners are identified and recruited, informed consent (Supplemental Material: Agreement form) will be obtained immediately.

### 2.5. Randomization and Blinding

Eligible facilities will be gathered through open solicitation. The joint research institutions will be selected based on discussions among and comprehensive review by the internal meetings of this association. We will assign the intervention group (facility) and the control group (facility) at a 1:1 ratio through stratified randomization according to the volume of potential patients. Patients from each facility who provide their consent will be included as intervention subjects. Since the study involves a behavioral intervention without a potential placebo counterpart, it will be impossible to blind both investigators and participants to group allocations. However, data collection research assistants will be trained on consistent administration of measures and awareness of biases.

### 2.6. Study Participants

The participants will be adults aged 18 years or older diagnosed with chronic HF who are classified as Stage C according to the ACCF/AHA HF staging system, and who have provided informed consent to participate in the study; the intervention group will receive in-person and phone-based care support program, and the control group will receive usual medical care and management at each healthcare facility.

### 2.7. Inclusion Criteria

Patients must have had one or more hospitalizations due to HF in the year preceding the index admission. Additionally, they must exhibit one or more of the following adherence-related issues:

- Living alone
- Deterioration in familial support structures
- Mental health concerns, including depression
- Cognitive impairment
- Complex medication regimens and instructions
- Experiencing adverse drug reactions or side effects
- Insufficient comprehension of medication regimen
- Inadequate understanding of the disease process and prognosis
- Challenges in managing medication administration, weight monitoring, and fluid balance
- Missing scheduled appointments (no-shows)

### 2.8. Exclusion Criteria

Patients are excluded who have impaired understanding or judgment due to factors which would make it difficult to obtain their consent to participate in the study.

## 3. Study Outcomes

### 3.1. Primary Outcomes

Self-care behavior improvement measured by EHFScBS. The Japanese version of the EHFScBS is a 12-item scale that uses a five-point response format, with lower total scores indicating better self-care behavior (range: 12–60); the questionnaire’s validity and the reliability of the Japanese version have been demonstrated [15].

### 3.2. Secondary Outcomes

Worsening HF, as evinced by the following:

- Incidence of readmission due to HF within 30 days, 3 months, 6 months, or 1 year after discharge;
- Duration of home care until readmission;
- Symptoms observed during unscheduled visits (prevention of symptom worsening through early visits);
- Changes in blood levels of BNP and NT-proBNP.

## 4. Interventions

**Figure 1:**
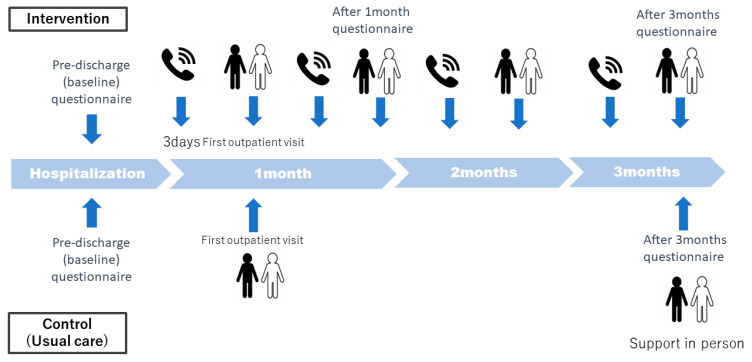
Schematic representation of the interventions in the intervention and control groups. This illustrates the frequency of interventions and the timing of questionnaire surveys in both the intervention and control groups. For the intervention group, there is a phone call on the third day, followed by face-to-face or phone interventions once a week for a month. After this initial month, both interventions continue once a month. In the control group, participants typically receive the usual care and are mostly asked to attend outpatient appointments two weeks later.

The intervention will be conducted in healthcare facilities that provide inpatient and outpatient care for chronic HF. The intervention will be carried out by HF nurses or nurses with at least 3 years of experience in HF nursing. The intervention period is set for three months after discharge, with a focus on the first month as a period of intensive support (Figure 1).

On the third day after discharge, support is provided via phone to the patient and their family to confirm their living situation, medication adherence, and self-management status related to weight and blood pressure control. If the patient was discharged to a facility, information is gathered from the facility staff and nursing consultation and guidance is provided to the patient and staff. In-person support for approximately 30 min is provided at the first outpatient visit after discharge (usually about 2 weeks later). For one month after discharge, support is provided once a week via in-person or phone methods. Following the initial month where support is provided weekly, both in-person and phone support will be offered once a month for the next three months. Depending on the patient’s condition and the judgment of the nursing staff, additional support may be provided as needed.

## 5. Control

During hospitalization, the usual medical care and discharge instructions according to the patient’s condition are provided, in the same manner as for the intervention group. In the outpatient setting, routine outpatient visits and care are also provided in each medical institution. The usual care is based on the concept of self-monitoring for HF patients and follows the disease-management program outlined within the heart failure guideline in Japan. Therefore, patient education during hospitalization is conducted appropriately, specifically including providing education to HF patients on self-monitoring their symptoms and other findings. However, regarding post-discharge care, even if the environment for structured nurse interventions is available, these are not implemented in the control group, in order to ensure a clear distinction between the intervention and usual care [19].

## 6. Data Collection

**Figure 2:**
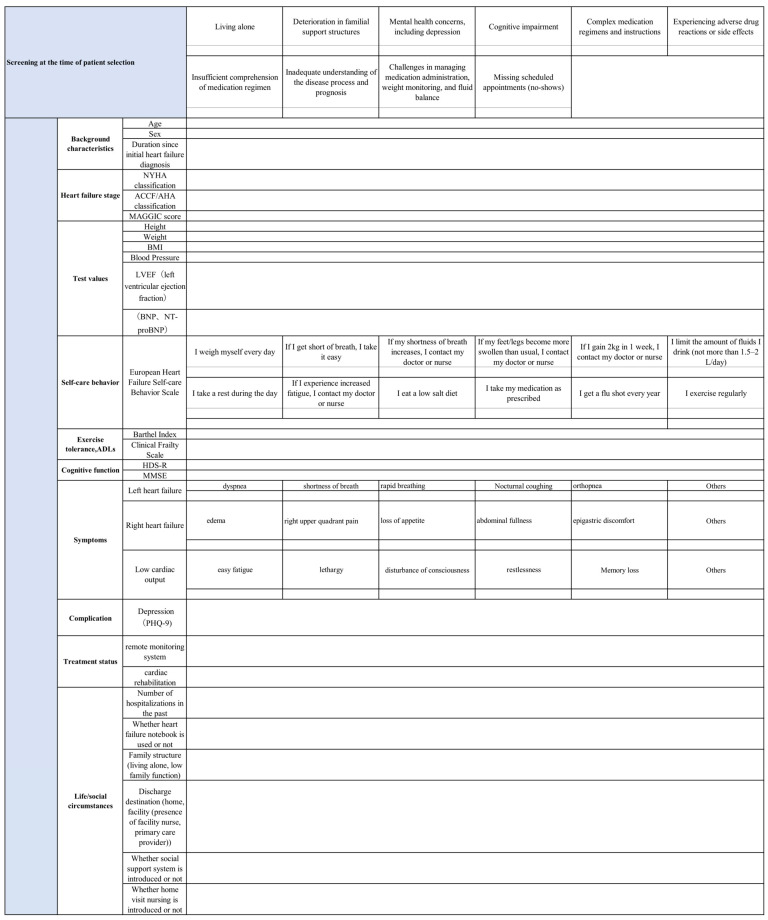
Baseline data collection sheet. This represents the pre-discharge data collection sheet, including the EHFScBS, European Heart Failure Self-Care Behavior Scale; NYHA, New York Heart Association; ACCF/AHA, American College of Cardiology Foundation/American Heart Association; MAGIC, Meta-Analysis Global Group in Chronic Heart Failure; BMI, body mass index; LVEF, left ventricular ejection fraction; BNP, brain natriuretic peptide; HDS-R, Revised Hasegawa’s dementia scale; MMSE, Mini Mental State Examination; and PHQ-9, Patient Health Questionnaire-9.

Baseline information will be collected prior to intervention (Figure 2). This includes age, gender, duration since initial HF diagnosis, HF stage according to NYHA (New York Heart Association) classification, ACC/AHA classification, and MAGIC (Meta-Analysis Global Group in Chronic HF) score. Test values include height, weight (variation), BMI, blood pressure, LVEF (left ventricular ejection fraction), BNP and NT-proBNP blood tests, and scores on the “EHFScBS” for self-care behavior. Exercise tolerance is assessed using the ADL and Frailty Index and Clinical Frailty Scale, and cognitive function is evaluated using HDS-R and MMSE. Physical findings include symptoms of left HF (shortness of breath, dyspnea, rapid breathing, nocturnal cough, and orthopnea), symptoms of right HF (edema, right hypochondrial pain, loss of appetite, abdominal bloating, discomfort in the epigastric region), and symptoms caused by low cardiac output (fatigue, weakness, altered consciousness, restlessness, and decreased memory). Depression is assessed using the PHQ-9 and information on treatment status is collected. The presence or absence of remote monitoring system implementation, cardiac rehabilitation, past hospitalization episodes, utilization of HF notebook, family structure (living alone, decreased family functioning), discharge destination (home or facility with or without a nurse and primary care provider), social support system, and introduction of visiting nursing are also collected. Additionally, qualitative measures to capture patient experiences and satisfaction with the intervention will be reported, including details on support content (conducted in-person or by phone, with specific support details recorded) and patient reactions (content of patient statements) will be collected.

In the intervention group, the same set of items as the baseline data, such as symptom control status, self-care behavior, self-reported symptoms and signs, and social circumstances will be collected at occurrence of readmission due to HF within 30 days or 3 months after discharge. The reason for readmission and duration of home care until readmission are collected. In addition, to capture the status of the intervention, the number of outpatient visits, including unscheduled visits, and the dates, times, and number of sessions by telephone and in-person are noted. If there are unscheduled teaching sessions, their content is also noted in the record. If the intervention has resulted in avoidance of hospitalization due to early visit or adjustment of oral medications, this should be reported.

In the control group, the same set of items as in the baseline data will be collected at 3 months after discharge. As with the intervention group, occurrence of readmission due to HF within 30 days or 3 months after discharge, and the reason for readmission and duration of home care until readmission are noted. Additionally, the number of outpatient visits (scheduled and unscheduled), and whether or not face-to-face medical guidance was provided are determined. If so, date, time, face-to-face time and content are collected.

## 7. Data Collection and Management

Data will be collected by the local researcher and nurses. Data will be coded and entered into either paper Case Record Forms or computer databases. Data entered on paper records will be sent directly to the Central Registration Center located at the Japanese Nursing Association. Data management will require two independent staff members in order to enter data into an Excel spreadsheet and have it saved onto a password-protected Universal Serial Bus mass storage device. Data management will also require double-checks for any potential errors in the input contents. Paper materials related to each participant’s evaluation will be stored in an evaluation binder, while intervention materials will be stored in a separate intervention binder. The binders holding evaluation and intervention forms will be kept inside a lockable shelf at Japanese Nursing Association and will be stored for up to 5 years after the examination ends.

## 8. Statistical Method

The Japan Nursing Association will submit the data received from the intervention and control groups’ medical institutions to a contracted data analysis organization for analysis. The between-group comparison of the change in self-care behavior continuity and changes in BNP and NT-proBNP in the baseline values at discharge and changes after intervention (1 and 3 months later) will be performed. The Spearman rank correlation coefficient will be used to measure the relationship between the scores of cardiac functions and the EHFScBS. The Mann–Whitney U-test will be used to compare the scores of the EHFScBS between the two groups with respect to patient backgrounds. The McNemar test will be used to analyze the changes in the two groups’ readmission and symptom deterioration rates, with the corresponding t-test employed for frequency, relative risk, and absolute risk reduction for risk difference. The presence or absence of readmission and the time to readmission between the intervention and control groups will be analyzed by the Kaplan–Meier method and tested for significance using the log-rank test. The Cox proportional hazard model and propensity score analysis will be used to compare between-group differences. Multiple imputation techniques will be employed to handle missing data, ensuring robustness and reducing bias in the findings. In the above analysis for the comparison, covariate adjustment from the baseline patient characteristics will be applied to gain efficiency in the estimation for the treatment effect, when it is necessary.

## 9. Ethics Approval and Dissemination

This study has been approved by the Japan Nursing Association Research Ethics Committee (Approval Code: 2022-10; Approval Date: 7 September 2022) and will be conducted with the consent of the participating institutions and patients/families. The following points will be explained orally and in writing, and agreement will have been obtained before the study is conducted. (1) Participation and cooperation in the study are voluntary for the cooperating institutions and the target patients, and neither will be disadvantaged by their participation or non-participation. (2) It is possible to stop participating or cooperating during the study, and this will not cause any disadvantages. (3) The data collected from the target patients will be processed to ensure that individuals cannot be identified, and the data will then be submitted to the Japan Nursing Association and the data analysts. The obtained data will be used as evidence for policy recommendations by the Japan Nursing Association (such as, e.g., the 2021 revision of medical fees, presentations at academic conferences, and opinions expressed at the Central Social Insurance Medical Council).

## 10. Discussion

The purpose of this clinical trial is to determine whether nurse-led structural interventions, particularly including early phone calls, improve self-care measures in heart failure patients and reduce heart failure-related readmissions and mortality.

Regarding nurse-led structural interventions in heart failure, Son et al. analyzed eight RCTs and demonstrated the effectiveness of interventions in reducing all-cause readmission (risk ratio [RR] = 0.75, 95% confidence interval [CI] = 0.66–0.85), heart failure-specific readmission (RR = 0.60, 95% CI = 0.42–0.85), and all-cause mortality or readmission (RR = 0.71, 95% CI = 0.61–0.82) [13]. However, they found that nurse-led heart failure self-care education was not associated with improvements in quality of life or heart failure knowledge. Similarly, Qiu et al. conducted a review of 15 studies (excluding those by Cockayne et al.), showing that rehospitalization (RR: 0.81, 95% CI: 0.74–0.88; *p* = 0.00001) and mortality (RR: 0.69, 95% CI: 0.56–0.86; *p* = 0.0009) were significantly lower among CHF patients assigned to nurse-led interventions [20]. Furthermore, a systematic review by Huang et al. indicated that nurse-led self-care interventions improved self-care maintenance (mean difference [MD]: 9.58, 95% CI: 5.96 to 13.20, moderate certainty of evidence), self-care management (MD: 12.08, 95% CI: 8.05 to 16.11, high certainty of evidence), and self-efficacy (standardized mean difference [SMD]: 0.98, 95% CI: 0.42 to 1.54, moderate certainty of evidence) [21]. Many interventions often limit their scope to patients with reduced left ventricular ejection fraction, and data on post-discharge structured interventions remain unclear. This study aims to provide a comprehensive assessment of the effects of nurse-led structural interventions on self-care behaviors in heart failure patients, without restricting the analysis to those with reduced LVEF.

Research on structured interventions focused on post-discharge telephone support for heart failure patients is limited, but the approach holds diverse potential benefits beyond self-care management, including consultation on post-discharge life issues. It is believed to be more efficient than in-person support. Inglis et al., in their Cochrane systematic review, demonstrated the effectiveness of structured telephone support in reducing all-cause mortality (RR 0.87, 95% CI 0.77 to 0.90) and heart failure-related hospitalizations (RR 0.85, 95% CI 0.77 to 0.93) [5,6]. Inouye et al. showed that automatic telephone follow-up 48 h post-discharge can identify high-risk patients for readmission among heart failure patients. Such structured interventions centered on post-discharge telephone calls are not limited to heart failure but are implemented across various domains [22]. Miller et al., through a retrospective analysis including non-heart failure cases in a single-center setting, demonstrated significantly lower readmission rates within 7 days of discharge and a trend toward lower rates within 30 days with telephone follow-up within 72 h post-discharge, which aligns with the approach adopted in this study [23]. This study aims to further evaluate the effectiveness of structured telephone interventions, with an initial call scheduled at 72 h post-discharge.

## Data Availability

No new data were created or analyzed in this study. Data sharing is not applicable to this article.

## Supplementary Materials

The following supporting information can be downloaded at: https://www.mdpi.com/article/10.3390/jpm14080832/s1, Supplemental Material Figure S1: Agreement form. Supplemental Material Figure S2: SPIRIT2022 Checklist: Items Recommended to be addressed in a clinical trial protocol and related documents.
