# Supplementary material for: Propelling Nurse-Led Structured Intervention to Enhance Self-Care among Patients with Chronic Heart Failure (PROACT-HF): A Cluster Randomized Controlled Trial Study Protocol
Source: J Pers Med. 2024 Aug 6;14(8):832. doi: 10.3390/jpm14080832 (PMC11355338; doi:10.3390/jpm14080832)
Supplement: Supplementary file 1 [file jpm-14-00832-s001.zip › jpm-3110555-supplementary Figure S1.pdf]

CONSENT FORM

Title of Project:

Participation in Supportive Care Assistance through In-Person and Telephone Nursing by Nurses in Outpatient Settings

Name of Researcher:

The committee of Japanese Nursing Association

Please check the box ☐ next to each item after receiving explanations and understanding the content.

1. Purpose of the Study ☐
2. Study Methods and Duration ☐
3. Voluntary Participation and Cooperation ☐
4. Right to Refuse Participation and Cooperation ☐
5. Protection of Privacy ☐
6. Methods for Protecting Personal Information ☐
7. Expected Benefits from Participating in the Study ☐
8. Potential Discomforts and Response Measures ☐
9. Compensation for the Participants ☐
10. Publication of Study Results ☐
11. Procedures During and after the Study ☐

I have received a comprehensive explanation regarding the content listed above and fully understand it. Consequently, I voluntarily agree to participate in this study

Date: ______________________________

Name of Participant:______________________________

Signature:______________________________

I hereby confirm that the aforementioned individual has voluntarily agreed to participate in the study.

Date:______________________________

Name of Researcher/Investigator:______________________________

Signature:______________________________
